# BCL-X_L_ overexpression promotes tumor progression-associated properties

**DOI:** 10.1038/s41419-017-0055-y

**Published:** 2017-12-13

**Authors:** Daniela Trisciuoglio, Maria Grazia Tupone, Marianna Desideri, Marta Di Martile, Chiara Gabellini, Simonetta Buglioni, Matteo Pallocca, Gabriele Alessandrini, Simona D’Aguanno, Donatella Del Bufalo

**Affiliations:** 1 Preclinical Models and New Therapeutic Agents Unit, Regina Elena National Cancer Institute, Via Elio Chianesi 53, 00144 Rome, Italy; 2 Institute of Molecular Biology and Pathology, National Research Council, Via Degli Apuli 4, 00185 Rome, Italy; 3 Pathology Unit, Regina Elena National Cancer Institute, Via Elio Chianesi 53, 00144 Rome, Italy; 4 SAFU Unit, Regina Elena National Cancer Institute, Via Elio Chianesi 53, 00144 Rome, Italy; 5 Thoracic Surgery Unit, Regina Elena National Cancer Institute, Via Elio Chianesi 53, 00144 Rome, Italy; 6 Present Address: Unit of Cell and Developmental Biology, Department of Biology, University of Pisa, S.S. 12 Abetone e Brennero 4, Pisa, Italy

## Abstract

By using human melanoma and glioblastoma cell lines and their derivative BCL-X_L_ overexpressing clones, we investigated the role of BCL-X_L_ in aggressive features of these two tumor histotypes. We found that in both models, BCL-X_L_ overexpression increased in vitro cell migration and invasion and facilitated tumor cells to form de novo vasculogenic structures. Furthermore, BCL-X_L_ overexpressing cells exhibited higher tumors sphere formation capacity and expressed higher levels of some stem cell markers, supporting the concept that BCL-X_L_ plays essential roles in the maintenance of cancer stem cell phenotype. BCL-X_L_ expression reduction by siRNA, the exposure to a BCL-X_L_-specific inhibitor and the use of a panel of human melanoma cell lines corroborated the evidence that BCL-X_L_ regulates tumor progression-associated properties. Finally, the vascular markers and the vasculogenic mimicry were up-regulated in the BCL-X_L_ overexpressing xenografts derived from both tumor histotypes. In conclusion, our work brings further support to the understanding of the malignant actions of BCL-X_L_ and, in particular, to the concept that BCL-X_L_ promotes stemness and contributes to the aggressiveness of both melanoma and glioblastoma.

## Introduction

A growing body of results supports the evidence that BCL-X_L_, and more in general BCL-2 family members, are not only key regulators of apoptosis, but also actively participate in the regulation of other vital cellular functions. As a consequence, limiting the oncogenic properties of the anti-apoptotic proteins of this family to their ability to oppose apoptosis is an old concept. In particular, several pieces of evidence indicate that BCL-X_L_ elicits new functions, which are genetically distinct from its effect on apoptosis^1–3^. In particular, a pivotal role for BCL-X_L_ in vitro and in vivo invasion of malignant glioma^2^, colorectal carcinoma^4^, and breast carcinoma^1, 5^ has been described. Moreover, gain-of-function studies in models of pancreatic cancer, demonstrated accelerated tumor formation and growth, while genetic ablation of BCL-X_L_ attenuates invasiveness without affecting apoptosis or tumor growth^5,6^. BCL-X_L_ ability to induce epithelial–mesenchymal transition has been also described together with the relevance of BCL-X_L_ nuclear localization in this phenomenon^5,7^. In fact, several reports indicate that BCL-X_L_ and other antiapoptotic proteins also reside in the nuclear membrane, even if they are primarily localized in the outer mitochondrial membrane, and they may even function within the nucleus, binding nuclear proteins and modulating the transactivity of several transcription factors^8–11^. However, BCL-X_L_ overexpression is not always sufficient for inducing its effects on tumor progression, and additional treatments may be necessary in some cases^6^.

We previously identified a novel function of BCL-X_L_ in promoting tumor angiogenesis through the nuclear factor kappa B (NF-kB)/interleukin 8 (CXCL8) axis in tumor cell lines with a different origin, including glioblastoma and melanoma^12–14^. The ability of BCL-X_L_ protein to modulate the angiogenic potential of cancer cells has been confirmed by using antisense oligonucleotides^15,16^. Our results are consistent with studies showing that both BCL-X_L_ and BCL-2 are key regulators of the angiogenic crosstalk between tumor and neovascular endothelial cells^17,18^.

Recent advances also highlighted a role for BCL-X_L_ in cancer stem cells (CSC) biology of different tumors: survival of tumors including lung and colon carcinoma has been shown to depend primarily on BCL-X_L_^5,19,20^. Moreover, the inhibition of BCL-X_L_ protein expression and the increased responsiveness of patient-derived glioblastoma and colon stem-like cells have been reported after treatment with BCL-2 family inhibitors^20,21^. BCL-X_L_ protein activation is also a central molecular mechanism by which senescent cells acquire increased resistance to apoptosis, and the block of BCL-X_L_ specifically induces apoptosis of senescent cells both in vitro and in vivo^22^.

BCL-X_L_ is frequently overexpressed, in comparison with normal tissue counterparts, in a significant subset of common cancers, including melanoma and glioblastoma. In particular, BCL-X_L_ expression increases during melanoma progression from primary to metastatic melanoma^23^. Moreover, one of the primary means by which melanoma cells evade apoptosis induced by different stimunli, is by up-regulation of anti-apoptotic proteins, including BCL-X_L_. Furthermore, the application of BCL-X_L_/BCL-2 inhibitors induces apoptosis in melanoma cells at different clinical stages including melanoma-initiating cells^23–25^. Members of the BCL-2 family are crucial regulators of cell death also in glioblastomas and the anti-apoptotic family members, including BCL-X_L_, are often overexpressed in this neoplasia^2,26^. Moreover, BCL-X_L_ levels are related to the sensitivity of glioblastoma cells to anti-neoplastic treatments^21,27^.

In this study, we investigated the functional role of BCL-X_L_ overexpression in aggressive features of melanoma and glioblastoma. We provide evidence that in both tumor histotypes BCL-X_L_ modulation regulates in vitro cell migration and invasion, and the ability of tumor cells to form de novo vasculogenic structures. Furthermore, BCL-X_L_ overexpressing cells exhibited higher CSC phenotype. Finally, even if no difference was observed in in vivo tumor growth, the expression of the vascular markers and the vasculogenic mimicry (VM) were up-regulated in the BCL-X_L_ overexpressing xenografts.

## Results

### BCL-X_L_ overexpression increases in vitro cell migration and invasion and promotes capillary-like structure formation

**Fig. 1:**
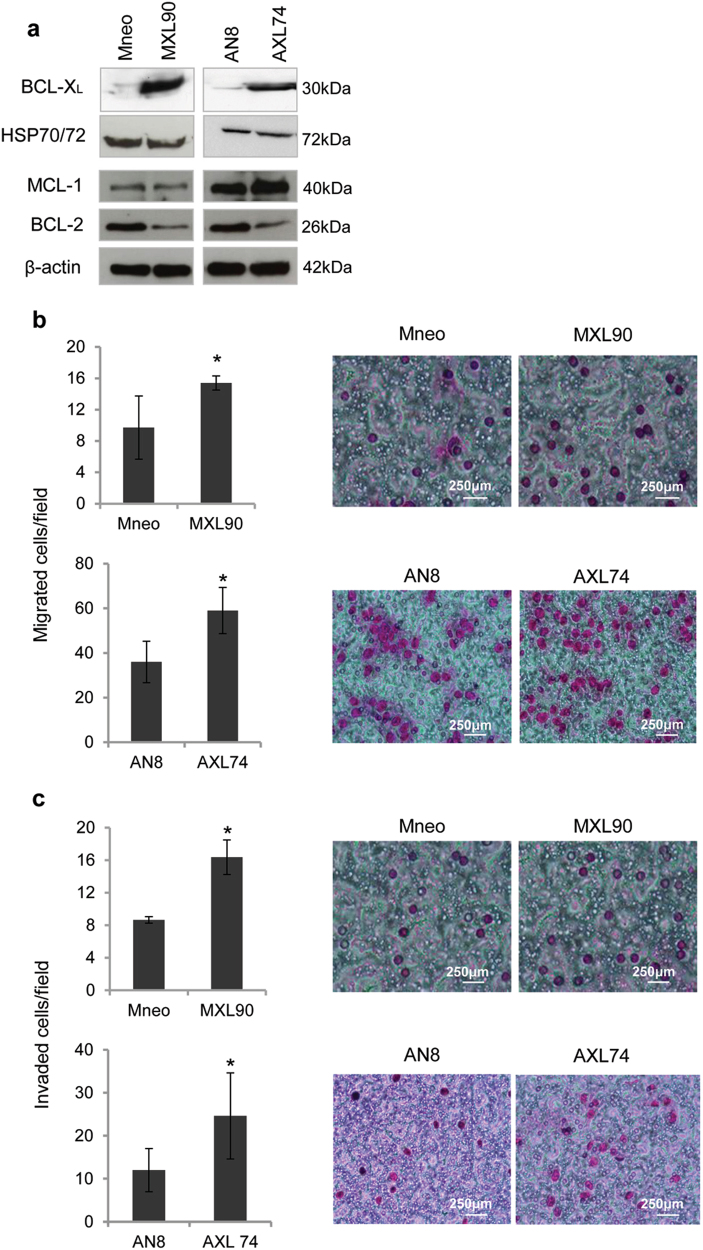
BCL-X_L_ overexpression promotes in vitro cell migration and invasion **a** Western blotting analysis of BCL-X_L_, BCL-2, and MCL-1 expression in melanoma control (Mneo), glioblastoma control (AN8), melanoma overexpressing BCL-X_L_ (MXL90) and glioblastoma overexpressing BCL-X_L_ (AXL74) cells. Reported images are representative of three independent Western blotting with similar results. HSP70/72 and β-actin expression was evaluated to confirm equivalent transfer and loading. Quantification and representative images of in vitro cell (**b**) migration and (**c**) invasion of Mneo, AN8, MXL90 and AXL74 cells. **b**, **c** Data were expressed as average ± standard deviation. *p < 0.05 after applying Student’s *t*-test

To evaluate whether BCL-X_L_ overexpression promotes tumor progression-associated properties, we used control and BCL-X_L_ overexpressing clones generated from human melanoma M14 (Mneo, MXL90) and ADF glioblastoma (AN8, AXL74) cells^13^ (Fig. 1a). We performed Western blot analyses to evaluate the expression of other pro-survival proteins, such as BCL-2 and MCL-1, in these clones. As reported in Fig. 1a, while BCL-X_L_ overexpressing clones show superimposable expression level of MCL-1 protein when compared to control clones, a reduced expression of BCL-2 protein was observed in both melanoma and glioblastoma models. We next confirmed the anti-apoptotic function of BCL-X_L_. As reported in Supplementary Fig. S1, BCL-X_L_ overexpression protects from staurosporine (STR)-induced and cisplatin (DDP)-induced apoptosis in both models. During in vitro cell proliferation, activation of apoptosis can occur due to depletion of nutrients or survival factors from the culture media. Thus, we also analyzed if apoptosis occurs in control transfectants during the in vitro growth and if BCL-X_L_ overexpression protects from eventual-induced apoptosis. As reported in Supplementary Fig. S2, no differences between control and BCL-X_L_ overexpressing clones were observed in terms of apoptosis activation. In particular, the Annexin V/PI staining, performed from 6 to 120 h after plating the cells, demonstrated a percentage of apoptotic cells lower than 9% both in control and BCL-X_L_ overexpressing cells. Similarly, PARP cleavage, a classical apoptotic marker, was not evidenced either in control or BCL-X_L_ overexpressing cells at the different time points analyzed. PARP cleavage was observed only in M14 and ADF control cells exposed to DDP (20 µg/ml, 24 h), which was used as positive control of apoptosis induction.

**Fig. 2:**
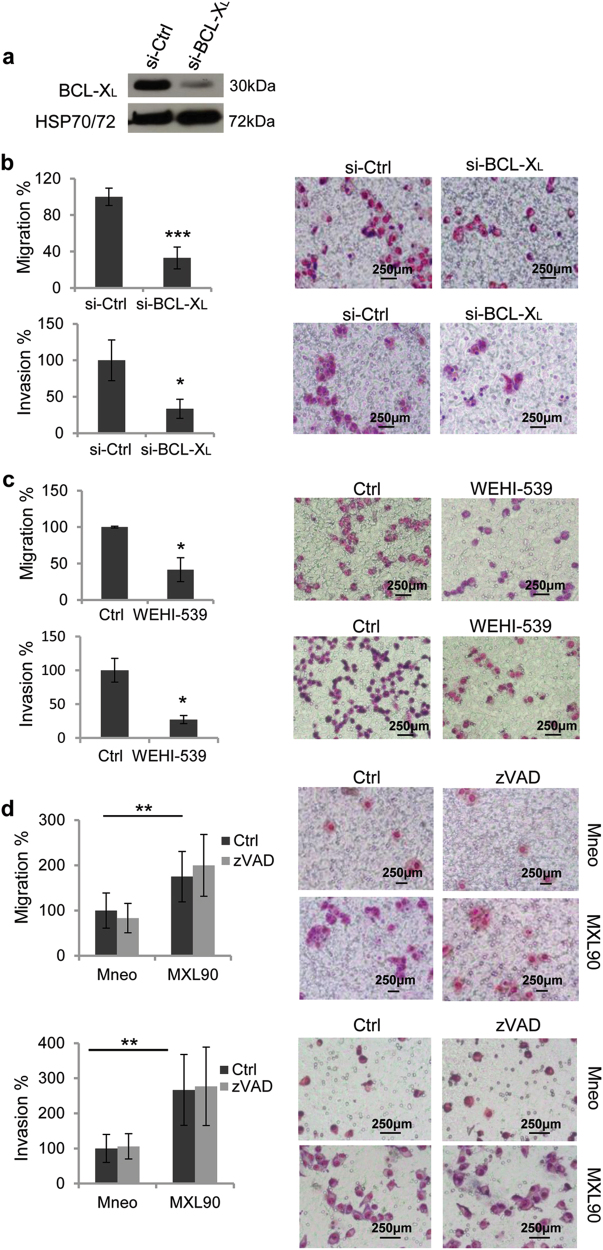
BCL-X_L_ downregulation reduces in vitro cell migration and invasion **a** Western blotting analysis of BCL-X_L_ expression in melanoma cells stably overexpressing BCL-X_L_ (MXL90) transfected with siRNA oligonucleotides against BCL-X_L_ (si- BCL-X_L_) or scramble (si-Ctrl) target sequences. HSP70/72 expression was evaluated to confirm equivalent transfer and loading. Reported images are representative of three independent Western blotting with similar results. Quantification and representative images of in vitro cell migration and invasion of (**b**) MXL90 cells transfected with si-BCL-X_L_ or si-Ctrl, (**c**) MXL90 cells exposed to 20 μM WEHI-539, (**d**) melanoma control (Mneo) and BCL-X_L_ overexpressing (MXL90) cells in presence of 50 μM pan caspase inhibitor z-VAD-FMK (zVAD) or DMSO (Ctrl). **b**–**d** Values are expressed as a percentage of migrated/invaded cells with respect to control. Data were expressed as average ± standard deviation. *p < 0.05, **p < 0.01, ***p < 0.001 after applying Student’s *t*-test

Next, to ascertain whether BCL-X_L_ overexpression affects cell migratory and/or invasive ability, we performed in vitro transwell migration and invasion assays. The number of invaded and migrated cells was significantly higher in cells overexpressing BCL-X_L_ when compared to control cells, thus indicating that BCL-X_L_ overexpression leads to an increase of the migratory and invasive capacity of both melanoma and glioblastoma cells (Fig. 1b, c). We confirmed the role of BCL-X_L_ in regulating melanoma cell migration and invasion in RNA interfering (siRNA) experiments aimed at lowering BCL-X_L_ levels in a representative BCL-X_L_ overexpressing melanoma clone (Fig. 2a). As depicted in Fig. 2b, BCL-X_L_ downregulation decreased both migration and invasion of about 60%, when compared to cells transfected with control siRNA. Treatment of melanoma BCL-X_L_ overexpressing clone with the BCL-X_L_ selective inhibitor WEHI-539^28^ corroborated the experiments with BCL-X_L_ siRNA knockdown: a significant reduction of both migration and invasion was observed after treatment with WEHI-539 (Fig. 2c). Similar results, in terms of reduction of both migration and invasion properties, were observed when a glioblastoma clone overexpressing BCL-X_L_ protein was treated with WEHI-539 (Supplementary Fig. S3).

To exclude that the increased migration and invasion observed in BCL-X_L_ transfectants might be related to protection from apoptotic program eventually activated during the assays, we also evaluated migration and invasion in the presence of z-VAD-FMK (zVAD). This irreversible pan-caspase inhibitor used at the dose of 50 μM, demonstrated to block apoptosis induced by DDP (Supplementary Fig. S1). As reported in Fig. 2d, the addition of zVAD did not affect the ability of cells to migrate and invade, thus demonstrating that apoptosis was not induced in control clone during the assay. These results also evidenced that the ability of BCL-X_L_ to increase migration and invasion was not related to protection from apoptosis activated during the assays.

**Fig. 3:**
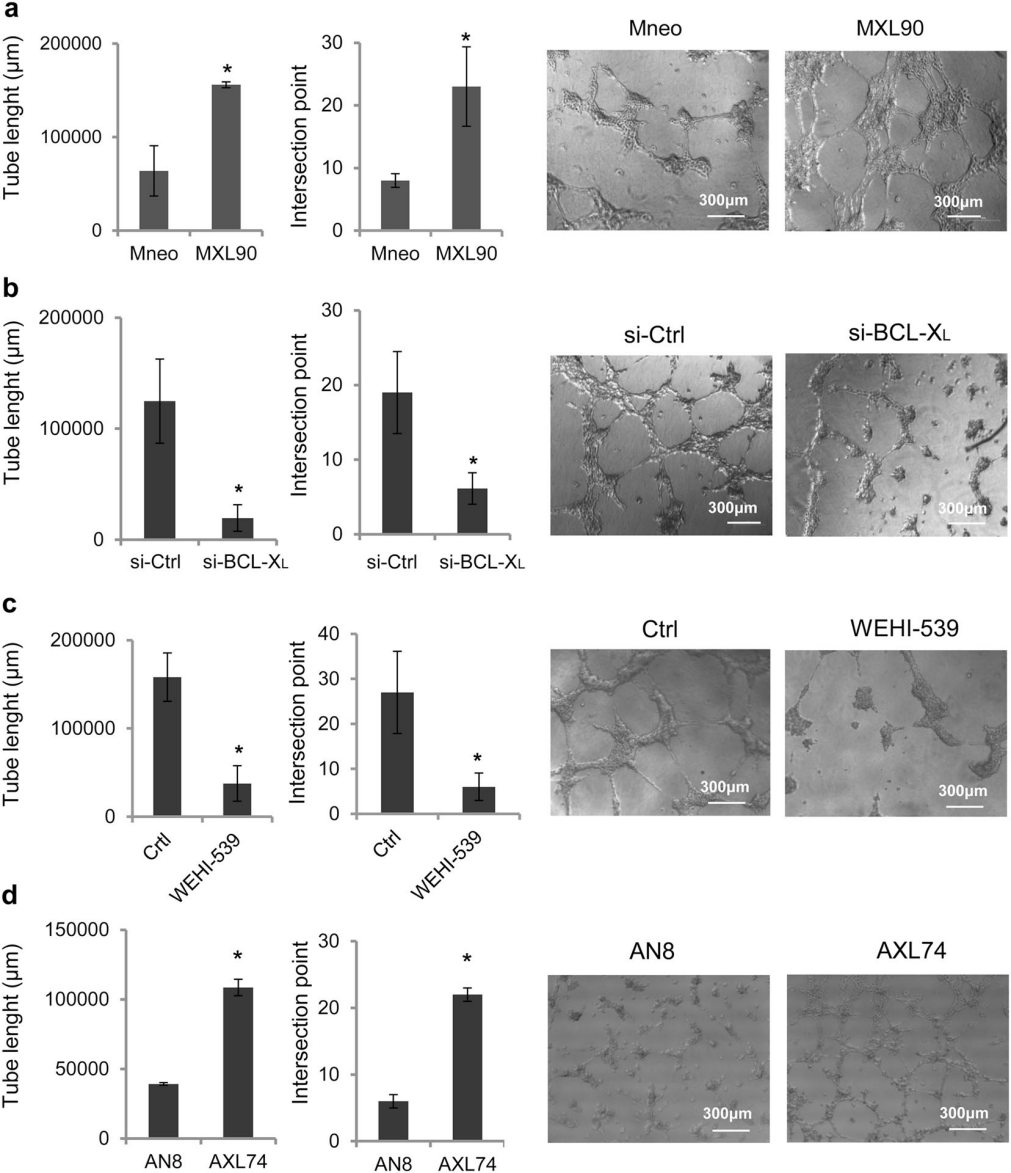
BCL-X_L_ modulates capillary-like structure formation Quantification (tube length and number of intersection point) and representative images of capillary-like structure formation in (**a**) melanoma control (Mneo) and BCL-X_L_ overexpressing (MXL90) cells, (**b**) MXL90 transfected with siRNA oligonucleotides against BCL-X_L_ (si-BCL-X_L_) or scramble (si-Ctrl) target sequences, (**c**) MXL90 treated with 20 μM WEHI-539, (**d**) glioblastoma control (AN8) and BCL-X_L_ overexpressing (AXL74) cells. Cells were plated on basement matrix extracts. Experiments have been performed under normoxic condition. Data were expressed as average ± standard deviation. *p < 0.05 after applying Student’s *t*-test

We further investigated the potential role of BCL-X_L_ on VM, the formation of vascular channels seeding melanoma or glioblastoma cells in serum-free medium onto the gelled basement matrix extracts (BME). VM formation is an alternative way to provide sufficient blood perfusion for highly malignant solid tumors^29,30^. As reported in Fig. 3, BCL-X_L_ overexpressing clones from both melanoma (Fig. 3a) and glioblastoma (Fig. 3d), demonstrated an enhanced VM, evaluated in terms of both tube length and number of intersections, when compared to the respective corresponding control clones. Downregulation of BCL-X_L_ in MXL90 cells by specific siRNA (Fig. 3b) or by BCL-X_L_ inhibitor WEHI-539 (Fig. 3c), resulted in serious impairment of VM in BCL-X_L_ overexpressing melanoma cells, when compared to control cells. A significant reduction of VM was also observed in glioma BCL-X_L_ overexpressing cells treated with BCL-X_L_ inhibitor WEHI-539 (Supplementary Fig. S3).

### BCL-X_L_ overexpression promotes CSC phenotype

**Fig. 4:**
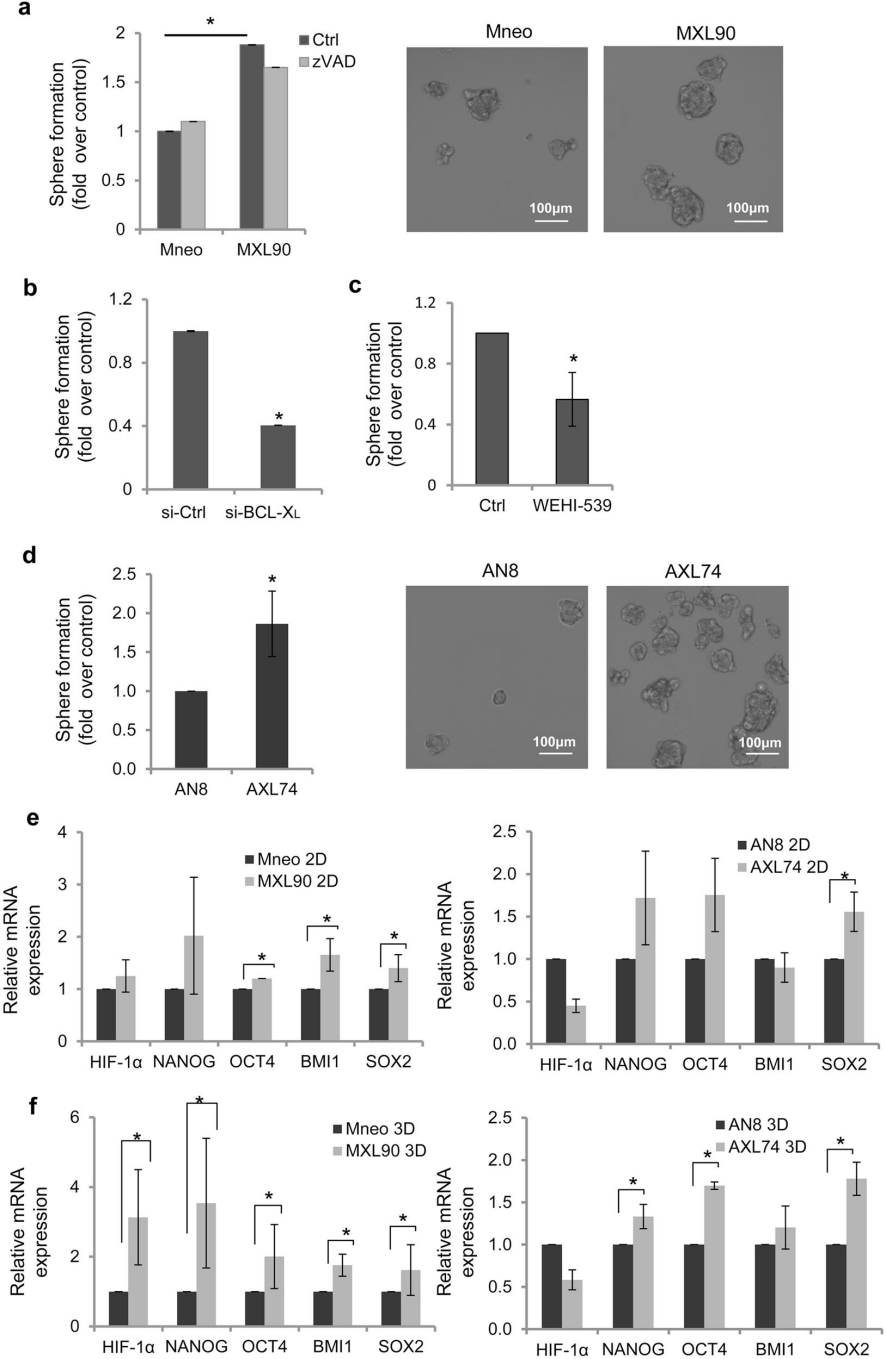
BCL-X_L_ modulates cancer stem cell phenotype Quantification and representative images of tumor sphere-forming capacity by (**a**) melanoma control (Mneo) and BCL-X_L_ overexpressing (MXL90) cells treated with 50 μM zVAD or DMSO (Ctrl), and (**d**) glioblastoma control (AN8), and BCL-X_L_ overexpressing (AXL74) cells. (**b**, **c**) Quantification of tumor sphere-forming capacity by MXL90 cells (**b**) transfected with siRNA oligonucleotides against BCL-X_L_ (si-BCL-X_L_) or scramble (si-Ctrl) target sequences, or **c** treated with 20 μM WEHI-539. Data shown represent the fold of spheroids formation over control. (**e**, **f**) Quantitative real-time polymerase chain reaction analysis of HIF-1α, NANOG, OCT4, BMI1, and SOX2 mRNA in Mneo, MXL90, AN8, and AXL74 cells grown in (**e**) adherent condition (2D) or (**f**) as tumor spheroids (3D). Values are expressed as means of ratio ± standard deviation, where ‘ratio’ was calculated considering BCL-X_L_ overexpressing cells vs. control cells. **a–f** *p < 0.05 after applying Student’s *t*-test

CSC have been demonstrated to transdifferentiate into different phenotypes and to form vessel-like networks, ‘mimicking’ the pattern of embryonic vascular networks^31,32^. In addition, they have been shown to be associated with tumor growth, local invasion, and distant metastasis, and to drive response to therapy^33^. Thus, to analyze the possible involvement of BCL-X_L_ in promoting stemness in our models, we firstly evaluated in vitro tumor sphere formation by plating tumor cells in the permissive medium. We evidenced that BCL-X_L_ overexpression promoted 3D spheroid formation both in melanoma (Fig. 4a) and glioblastoma (Fig. 4d) models. At the end of established experimental period (10 days) both melanoma control and BCL-X_L_ overexpressing cells showed more than 80% of viability (Fig. S4), thus we can exclude activation of apoptosis in control clone during the 10 days of assay. We can also exclude that in BCL-X_L_ overexpressing clones the observed stemness phenotype is due to BCL-X_L_ canonical anti-apoptotic function. This evidence is confirmed by analysis of 3D spheroid formation in presence of zVAD. In fact, as reported in Fig. 4a, zVAD did not affect the ability to form 3D spheroids of both control and BCL-X_L_ overexpressing cells. In line with these results, mRNA expression of apoptosis-regulating proteins, such as BCL-X_L_, BCL-2, BAX, PUMA, NOXA, and BIM was not significantly modulated in cells grown as 2D and 3D spheroids (Supplementary Fig. S5).

The role of BCL-X_L_ in promoting 3D spheroid formation was confirmed in siRNA experiments. In particular, downregulation of BCL-X_L_ by specific siRNA (Fig. S4) resulted in the reduced 3D spheroid formation in melanoma model when compared to cells transfected with control siRNA (Fig. 4b). The reduced 3D spheroid formation was also observed in both melanoma (Fig. 4c) and glioma (Fig. S3) BCL-X_L_ overexpressing clones after treatment with WEHI-539.

Notably, a similar percentage of cell viability was found in control cells, silenced or WEHI-539 treated melanoma BCL-X_L_ overexpressing clone (Fig. S4), thus demonstrating that the observed reduction of 3D spheroid formation after genetic or pharmacological inhibition of BCL-X_L_ was not due to apoptosis induction.

Next, we also evaluated the modulation of several CSC markers in terms of mRNA expression, including HIF-1α, NANOG, OCT4, BMI1, and SOX2 in cells grown under adherent (2D, Fig. 4e) or spheroid (3D, Fig. 4f) conditions. An enhancement in the expression of different stem cell markers was observed in BCL-X_L_ transfectants from both models grown as 3D spheroids when compared to control clones cultured in the same condition. Interestingly, when cells were grown in 2D, the expression levels of OCT4, BMI1, SOX2 for melanoma cells and of SOX2 for glioblastoma were modulated by BCL-X_L_ overexpression (Fig. 4e). Taken togheter, these results indicate that BCL-X_L_ increases CSC features.

**Fig. 5:**
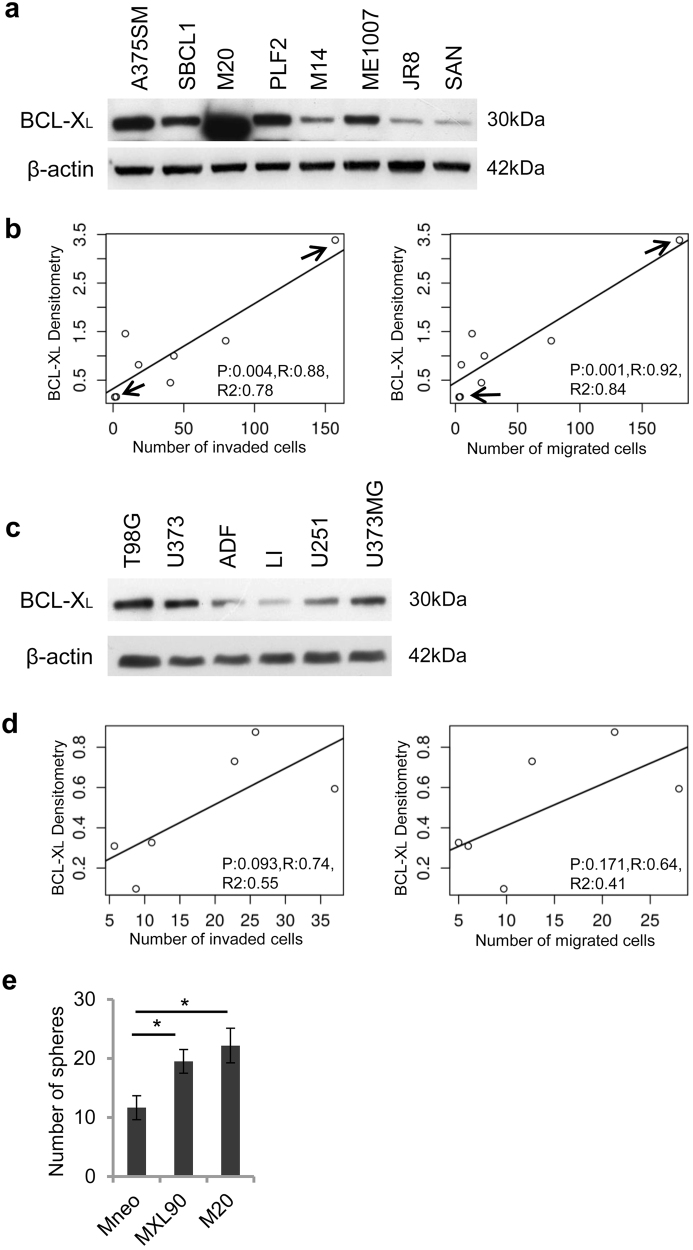
BCL-X_L_ protein expression correlates with invasion and migration in a panel of melanoma cell lines Western blotting analysis BCL-X_L_ protein expression in (**a**) melanoma and **(c)** glioblastoma cell lines. Reported images are representative of two independent experiments with similar results. β-actin expression was evaluated to confirm equivalent transfer and loading. (**b**, **d**) Correlation among BCL-X_L_ evaluated via Western blotting analysis and invasion/migration properties in a panel of melanoma (**b**) and glioblastoma (**d**) cell lines. Densitometric values of normalized BCL-X_L_ protein level were plotted against the number of migrated/invaded cells. Arrows indicate cell lines with highest (M20) and lowest (JR8, SAN) migration/invasion properties. JR8 and SAN cells showed superimposable values. Results were considered to be statistically significant if *p  < 0.05. **e** Tumor sphere-forming capacity evaluated in Mneo, MXL90 and M20 cells. Data were expressed as average ± standard deviation

We also evaluated in vitro cell invasion/migration and stemness properties of a panel of both melanoma and glioma cell lines with variable levels of endogenous BCL-X_L_ protein. Western blot analysis indicates that BCL-X_L_ protein was detectable in all the cell lines analyzed, even though to a different extent (Fig. 5a, c). Furthermore, we performed an exploratory linear regression analysis, correlating BCL-X_L_ protein level with the number of migrated and invaded cells across all cell lines representing each tumor histotype. As evidenced in Fig. 5b, d and Supplementary Fig. S6, a positive correlation trend was found in all cases (R2 from 0.4 to 0.8), but the test was significant only for the melanoma cell lines (*p* < 0.05) (Fig. 5b). In particular, M20 cells expressing a higher level of BCL-X_L_ protein show higher invasive and migratory ability when compared to JR8 and SAN cells expressing lower levels of BCL-X_L_ protein. The missing 5–6% confidence from the correlation test in glioblastoma could be recovered with more samples.

Interestingly, M20 showed also tumors sphere formation ability similar to that observed in BCL-X_L_ overexpressing clone (Fig. 5e).

To investigate if BCL-X_L_ ability to promote tumors sphere formation is common to other anti-apoptotic members, we also performed experiments by using M14 melanoma control and BCL-2 overexpressing clones^34^. As reported in Supplementary Fig. S7, BCL-2 overexpression promoted the 3D spheroid formation. Moreover, as observed for BCL-X_L_ overexpressing cells, the addition of zVAD did not affect the tumor sphere forming ability of these cells. Pharmacological or genetic targeting of BCL-2 by the specific inhibitor ABT-199^35^ or a specific siRNA, respectively, caused a significant decrement of tumors sphere formation ability (Supplementary Fig. S7). Although the addition of zVAD to ABT-199 treated cells was sufficient to recovery from apoptosis induction, the presence of zVAD did not restore the sphere forming ability of BCL-2/6 (Supplementary Fig. S7). This result indicates that the reduced sphere formation cannot be due to apoptosis.

### BCL-X_L_ overexpression affects HIF-1, VEGF, and MMP2 expression in melanoma cells

To further explore the molecular pathway involved in BCL-X_L_-mediated tumor aggressiveness, we next focused our attention on HIF-1/VEGF axis, a key pathway involved in melanoma and glioblastoma vascularization and aggressiveness^31,36–40^. Although no effect of BCL-X_L_ overexpression on HIF-1/VEGF pathway was observed under the normoxic condition, interestingly we found that under hypoxia, BCL-X_L_ overexpressing melanoma cells showed a higher level of the α subunit of transcription factor HIF-1 respect to control transfectants grown under the same condition (Supplementary Fig. S8). Notably, BCL-X_L_ was able to increase HIF-1α expression also in other conditions strictly dependent on oxygen availability, as high cell density, a condition creating a local pericellular hypoxic microenvironment (data not shown). In agreement with these results, BCL-X_L_ overexpressing melanoma cells produced in their conditioned media compared to that from control clone, significantly higher level of VEGF protein, a HIF-1-dependent pro-angiogenic factor. Moreover, when BCL-X_L_ transfectants were compared to control ones, exposure to hypoxia also induced an increase in VEGF promoter and HIF-1 transcriptional activity (Supplementary Fig. S8).

We also demonstrated increased VEGF secretion and HIF-1α expression in low passage number of control and BCL-X_L_ stably transfected clones obtained from JR8 human melanoma parental cells, thus excluding that during long-term culture of tumor cells there was selection for specific subpopulations of cells (Supplementary Fig. S8). It is well known that metalloproteinases (MMP) are regulated by HIF-1 and that are involved in the regulation of the extracellular matrix degradation, required for cell migration, invasion, and VM. Thus, we analyzed MMP2 activation in melanoma clones under normoxia and hypoxia. As depicted in Supplementary Fig. S8, an enhancement of MMP2 expression was observed after BCL-X_L_ overexpression under normoxic conditions. MMP2 expression was also further increased when BCL-X_L_ overexpressing cells were exposed to hypoxia. Surprisingly, after exposure to hypoxia, no significant differences were observed after BCL-X_L_ overexpression in glioblastoma model, in terms of both VEGF protein secretion and HIF-1α protein stabilization (data not shown).

### Vascularization and VM are enhanced in BCL-X_L_ overexpressing tumors

**Fig. 6:**
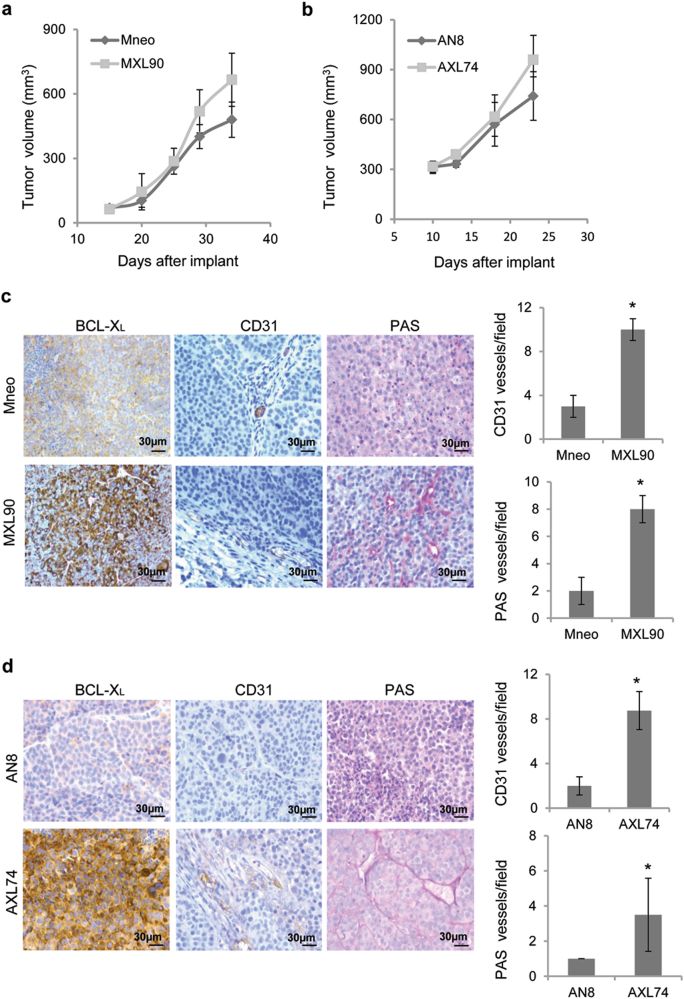
Vascularization is enhanced in BCL-X_L_ overexpressing tumors Tumor growth evaluation after injection in nude mice of (**a**) melanoma control (Mneo) and BCL-X_L_ overexpressing (MXL90) cells, and (**b**) glioblastoma control (AN8) and BCL-X_L_ overexpressing (AXL42) cells. Immunohistochemical analyses of BCL-X_L_ expression and vascularization in tumors obtained (**c**) 34 days after injection of melanoma cells, and (**d**) 23 days after injection of glioblastoma cells. Vascularization was evaluated by detecting CD31-positive endothelial cells and by Periodic acid-Schiff (PAS) staining. Data were expressed as average ± standard deviation

To corroborate our in vitro results, we established xenograft tumor models using melanoma or glioblastoma cells. As reported in Fig. 6a, b, BCL-X_L_ expressing clones did not show a significant induction of tumor growth, when compared to control ones. Histological analysis of tumor xenografts section revealed that the overexpression of BCL-X_L_ was stable during in vivo growth of melanoma and glioblastoma (Fig. 6c, d). More importantly, tumor xenografts sections from control tumors had a reduced number of CD31-positive (indicative of endothelial vessels) and Periodic acid-Schiff (PAS)-positive vessels (indicative of tumor vessels) when compared to tumor sections from BCL-X_L_ overexpressing xenografts. Consistent with the in vitro observations, these data showed that BCL-X_L_ enhances tumor angiogenesis in xenograft tumor models of both melanoma and glioblastoma.

## Discussion

Our current investigation provides additional, complementary data to previous ones, from our and other groups, in regard to the emergent function for BCL-X_L_ in tumor aggressiveness^1,2,4,5^. In particular, we demonstrated that exogenous BCL-X_L_ overexpression plays a powerful role in controlling multiple malignant properties of human melanoma and glioblastoma, including migration, invasion, and tumor cell plasticity. Our results also evidence BCL-X_L_ involvement in melanoma and glioblastoma stemness. Moreover, experiments performed with specific siRNA or with a specific BCL-X_L_ inhibitor, corroborated a direct effect of BCL-X_L_ on the observed phenotypes. The use of a panel of melanoma cell lines showing variable expression level of endogenous BCL-X_L_ protein corroborated the observations made in the engineered cell lines indicating clinical and pathophysiological relevance to cancer that express a high level of BCL-X_L_, including melanoma.

Our observations support the view that BCL-X_L_ works not only as essential cues for cell fate determination, but it also regulates pathways involved in the progression of melanoma and glioblastoma, as recently reported for pancreatic neuroendocrine and breast tumors^5^. The concomitant increase in invasion, migration, VM and CSC-like stemness pathways after BCL-X_L_ overexpression observed in our models, suggests that BCL-X_L_ protein may be an important in vivo environmental cue promoting melanoma and glioblastoma aggressiveness and CSC maintenance. BCL-X_L_ ability to induce these phenomena, together with the evidence of a correlation between invasive/migratory ability and the levels of endogenous BCL-X_L_ expression, emphasizes its relevance in regulating tumor aggressiveness.

Studies carried out with low passage number of BCL-X_L_ overexpressing clones excluded that during long-term culture there was a selection for a specific subpopulation of cells.

We also provide additional results to our previous ones, regarding the role of BCL-2 in tumor invasion, migration, and metastatization^34,37,38,41^ by demonstrating BCL-2 ability to promote CSC phenotype in melanoma. In agreement with previously reported data showing that breast, colon, lung, and leukemia CSC are especially vulnerable to BCL-2 family inhibitors^25^, our findings highlight that inhibition of anti-apoptotic BCL-2 family members could represent a promising approach to target chemotherapy-resistant CSC and plasticity of glioblastoma and melanoma.

We also found that BCL-X_L_ overexpression modulates HIF-1 and its target genes in melanoma models. While some anti-apoptotic proteins, including BCL-X_L_, have been identified as HIF-1 target genes^42–44^, this is the first evidence demonstrating HIF-1 modulation by BCL-X_L_ in a melanoma model. Similarly, we previously showed that under hypoxia BCL-2 promotes HIF-1-mediated VEGF expression in melanoma and breast carcinoma^41,45^.

Overall, our results demonstrate that in addition to their effect on the apoptotic pathway, both BCL-2 and BCL-X_L_ share the ability to regulate new functions. We have previously demonstrated that both BCL-2 and BCL-X_L_ proteins^11–15,37^ positively regulate invasion and migration and production of angiogenic factors in melanoma models. In this paper, we report that the increased invasion and migration induced by BCL-X_L_ is not related to other anti-apoptotic proteins, such as BCL-2 or MCL-1. In fact, forced BCL-X_L_ expression does not determine a modulation of MCL-1 protein, and even downregulation of BCL-2 protein in our melanoma models.

Our results demonstrating BCL-X_L_ ability to modulate the expression of a transcription factor, such as HIF-1 are in agreement with previous papers demonstrating BCL-X_L_ ability to exert transcriptional and epigenetic changes^5^. Experiments are ongoing to investigate the BCL-X_L_ localization at the nucleus in melanoma cells overexpressing BCL-X_L_ and the mechanism of BCL-X_L_-mediated activation of HIF-1/VEGF axis.

We previously demonstrated that BCL-X_L_ also promotes tumor angiogenesis through the NF-kB/CXCL8 axis both in melanoma and glioblastoma models^12–14^. Since the increased level of CXCL8 in breast CSC have been shown to contribute to breast cancer aggressiveness by promoting VM^46^, it is reasonable that CXCL8 might contribute to BCL-X_L_ increased VM formation, invasiveness, and stemness of both tumor types. Moreover, the high VM observed after BCL-X_L_ overexpression might be due to enrichment in CSC population, which participate in VM formation by interacting with the vascular niche to shape the proper tumor microenvironment and by differentiating into endothelial cell-like tumor cells to constitute VM structures^29,32,47^. A better understanding of BCL-X_L_, and more in general of BCL-2 family members, will provide an insight into the molecular mechanism of tumor progression, how conventional chemotherapy selectively kills cancer cells, and why some cancers are more chemosensitive than others. Our results also underline the impact of targeting BCL-2 family members to kill heterogeneous tumors and to eliminate CSC-resistant population.

Further exploitation of our understanding of the BCL-2 family promises to offer improved predictive biomarkers for oncologists and improved therapies for patients with cancer.

## Materials and methods

### Cell lines and reagents

Human melanoma (JR8, SAN, M20, PLF2, ME1007, M14, A375SM, SBCL1) and glioblastoma (ADF, LI, T98G, U251, U373, U373MG) cell lines were cultured as previously reported^12,13^. Stable control (Mneo) and BCL-X_L_ overexpressing (MXL90) clones previously generated from M14 human melanoma cells, stable control (J8neo) and BCL-X_L_ overexpressing (J8XL8 and J8XL10) clones from JR8 human melanoma, and control (AN8) and BCL-X_L_ overexpressing (AXL74) clones previously generated from ADF human glioblastoma cells^13^ were used. These cells were cultured in the presence of 800 μg/ml geneticin (Euroclone, Milan, IT) in RPMI medium (Euroclone) containing 10% inactivated fetal bovine serum (FBS) (Hyclone, Thermoscientific, South Logan, UT), 2 mM l-glutamine (Euroclone), and antibiotics. Stable control (puro) and BCL-2 overexpressing (BCL-2/6) clones previously generated from M14 cells were cultured in 10% FBS RPMI medium in the presence of 1 mg/ml puromycin (puro, Sigma–Aldrich, St. Louis, MO)^34^. Pooled siRNA oligonucleotides against BCL-X_L_ or BCL-2 or scramble (si-Ctrl) target sequences were purchased from Dharmacon RNA Technologies (siGENOME SMARTpool, Lafayette, CO, USA). For siRNA transfection, cells were seeded and after 24 h transfected with 50 nM pooled oligonucleotides mixture by using jetPRIME (Polyplus Transfection, Sébastien Brant Illkirch FRANCE) following the manufacturer’s protocol. After 24 h, the medium was changed and BCL-X_L_ protein expression was assessed 48 h after silencing by Western blot analysis.

#### Flow cytometric analysis

Flow cytometric analysis (BD Accuri C6, BD biosciences) was performed to evaluate cell cycle distribution by propidium iodide (PI) staining and to quantify apoptotic cells by AnnexinV-FITC/PI staining as already described^48^. Cell viability of spheroids was assessed by AnnexinV-FITC staining.

#### Western blotting analysis

Antibodies directed to BCL-X_S/L_ (#sc-634, Santa Cruz Biotechnology, CA, USA), HIF-1α (#610959, BD Biosciences, San Diego, CA, USA), HIF-1β (#611079, BD Biosciences), MMP2 (#sc-10736, Santa Cruz Biotechnology), cleaved PARP (#AB3565, Millipore, Billerica, MA, USA), MCL-1 (#sc-12756, Santa Cruz Biotechnology) were used. HSP70/72 (#HSP01, Calbiochem, San Diego, CA, USA), β-actin (#A1978, Sigma), and HSP90 (#610419 BD Biosciences) antibodies were employed to confirm equivalent transfer and loading. Antibody binding was visualized by enhanced chemiluminescence method (Pierce ECL Plus Western Blotting Substrate, Thermoscientific) according to manufacturer’s specification. The densitometric evaluation was performed using Image J software and normalized with relative controls depending on the analysis.

#### ELISA

The level of secreted VEGF by cells cultured for 18 h under normoxia (21% oxygen) or hypoxia (less than 1% oxygen) was assayed by ELISA kit according to the manufacturer’s instructions (R&D Systems, Minneapolis, MN, USA). VEGF in the supernatants was normalized to the number of adherent cells.

#### Luciferase assay

To study VEGF promoter and HIF-1 transcriptional activity, 7 × 10^4^cells were seeded in triplicate in 24-well plates; 24 h later cells were transfected with a total of 1 μg of DNA/well using JetPRime reagent (Polyplus transfection, Illkirch, France) according to the manufacturer’s protocol. Three different constructs were used: (1) the VEGF1511 fragment of the VEGF promoter construct including 1175 bp of the promoter from the start site of transcription and 336 bp of untranslated mRNA; (2) the 385 bp deletion fragment (VEGF385), containing a similar binding site to that of the HIF-1, was generated from 1511 bp fragment by restriction digestion; (3) the HIF-1 plasmid consisting of a vector expressing luciferase under the control of 4× hypoxia responsive element. To normalize for transfection efficiency, the PEQ-176 plasmid (0.5 μg) was included in the transfections. Samples were collected 18 h after the induction of hypoxia and analyzed for luciferase and β-galactosidase activity. Relative luciferase expression was determined as a ratio of β-galactosidase activity. Results were reported as fold of normalized luciferase activity over the negative control (empty vector).

#### Cell migration and invasion assays

For cell migration assay, 1 × 10^5^ cells were seeded in serum-free media into the upper chamber of Transwell (Corning, Costar, New York, USA) containing 8 µm pore polycarbonate membrane. The lower well contained medium with 10% FBS. After 8 h of incubation at 37 °C, cells remaining on the top side of the membrane were removed using a cotton swab, and migrating cells were fixed, stained (Differential Quick Stain Kit, Dade Behring, Marburg, Germany), photographed and counted.

For cell invasion assay, 8 × 10^4^ cells were seeded in serum-free media into the upper chamber of CultreCoat^®^ 24-Well Medium BME Cell Invasion Inserts (Trevigen, Gaithersburg, MD, USA) having a polycarbonate membrane with 8 μm pores coated with a thin basement membrane. After 8 h of incubation at 37 °C, cells remaining on the top side of the membrane were removed, and invading cells fixed, stained, photographed, and counted. Fifty micromolar of zVAD (Sigma-Aldrich) was dissolved in DMSO (Sigma-Aldrich) and added to cells seeded in serum-free media into the upper chamber for both migration and invasion assay. Cell migration and invasion were also evaluated after pharmacological inhibition of BCL-X_L_: cells were treated with 20 μM of WEHI-539 (MedChemExpress, New Jersey, United States), a selective inhibitor of BCL-X_L_, for 24 h, trypsinized and subjected to cell migration and invasion assays.

#### Tumor sphere-forming capacity

1 × 10^3^ cells were plated in six-well ultralow attachment surface plate and cultured as previously described^48^. After 10 days, spheres were photographed and counted. In the case of silencing experiments, cells were plated after 48 h of silencing and tumor sphere-forming capacity was evaluated after 10 days. The capacity to form tumor sphere was also evaluated after 4 days in presence of WEHI-539 (20 μM), zVAD (50 μM), or ABT-199 (1 μM), a specific BCL-2 inhibitor (Apexbio, Houston, USA).

#### Quantitative real-time polymerase chain reaction (qRT-PCR) analysis

Total RNA was extracted from in vitro cultured cells using a Qiagen RNeasy Mini kit (Qiagen, Redwood City, CA, USA) according to the manifacturer’s instructions. Reverse transcription was performed using RevertAid Reverse Transcriptase (Thermoscientific). qRT-PCR was performed with a Gene-Amp 7900 sequence detection system (Applied Biosystems, Foster City, CA, USA), using the SYBR green dye detection method. The mRNA levels were normalized using glyceraldehyde 3-phosphate dehydrogenase (GAPDH). Primers used to analyze each gene were: HIF-1α (Fw:CCAGTTAGGTTCCTTCGATCAGT, Rv:TTTGAGGACTTGCGCTTTCA); BMI1 (Fw:ATGTGTGTGCTTTGTGAG, Rv:AGTGGTCTGGTCTTGTGAAC); SOX2 (Fw:CACCCCTGGCATGGCTCTT, Rv:GAGCTGGCCTCGGACTTGA); NANOG (Fw:AATACCTCAGCCTCCAGCAGATG, Rv:TGCGTCACACCATTGCTATTCTTC); OCT4 (Fw:TCCCATGCATTCAAACTGAGGT, Rv:CCCAAAAACCCTGGCACAA); GAPDH (Fw:TCCTGAGCTGAACGGGAAG, Rv:GGAGGAGTGGGTGTCGCTGT); PUMA (Fw: AAGTCAGGACTTGCAGGCGCG, Rv: TGGGTCCCAGTCAGTGTGTGT); NOXA (Fw: CGCTGACGACGTCCCAGCGTTT, Rv: CGAAGACGGCGTTATGGGAGC); BIM (Fw: CAGAGATATCGATCGCCCAAG, Rv: CAGAGATATGGATCGCCCAAG); BCl-2 (Fw:CTGCACCTGACGCCCTTCACC, Rv: CACATGACCCCACCGAACTCAAAGA); BAX (Fw: TCCCGGCTCTCTGATCCCCG, Rv: GGCTAGGGGAACGCTATATGC); BCL-X_L_ (Fw: TTGGATGGCCACTTACCTGAAT, Rv: AACCAGCGGTTGAAGCGTT). Student’s *t*-test was used and results were considered to be statistically significant if *p* < 0.05 (*).

#### Analysis of capillary-like structures (CLS)

Two hundred and fifty microliters of BME (12.7 mg/ml, Trevigen) were dropped onto each well of a 24-well plate and were allowed to solidify for 1 h at 37 °C in humidified 5% CO_2_ incubator. 2 × 10^5^ cells were seeded in serum-free medium onto the gelled BME and incubated at 37 °C for 18 h. Then, CLS formation was photographed using light microscopy and quantified by evaluating the tube length and counting the number of cell junctions in 10 sets of images for each clone. Each clone was analyzed in duplicate in three different experiments using image analysis program (Image J v.1.34s; http://rsb.info.nih.gov/ij/). CLS was also evaluated after treatment of cells with 20 μM of WEHI-539 for 24 h or after BCL-X_L_ silencing.

#### Tumor growth and immunohistochemical (IHC) analysis

Cells in exponential growth phase were harvested from the culture, washed, and resuspended in PBS and injected subcutaneously (s.c.) for melanoma cells or intramuscularly (i.m.) for glioblastoma cells, into nude mice at 5 × 10^6^ viable cells/mice. Tumor weight was monitored and calculated as previously reported^37^. Female CD-1 nude (nu/nu) mice, 6–8 weeks old and 22–24 g in body weight were purchased from Charles River Laboratories (Calco, Italy). All procedures involving animals and their care were authorized and certified by D.lgs 26/2014 (816/2015-PR del 11/08/2015) of the Italian Minister of Health. 34 or 23 days after melanoma or glioblastoma cell injection, respectively, tumors were removed, placed in 10% buffered formalin for 24 h, dehydrated, and embedded in paraffin. For each tumor, three different 5 μm paraffin sections were analyzed and examined by light microscopy. BCL-X_L_ expression was evaluated using BCL-X_L_ monoclonal antibody (clone H-5, Santa Cruz Biotechnology). Immunoreactions were revealed by Bond Polymer Refine Detection, a biotin-free, polymeric horseradish peroxidase (HRP)-linker antibody conjugate system on an automated autostainer (Bond^TM^ Max, Leica Biosystem, Milan, Italy). The IHC results for BCL-X_L_ were recorded as positive when tumor cells exhibited a strong homogeneous cytoplasmic immunoreaction, whereas cases with faint staining were regarded as negative (200× and 400× magnification). Vascularization was evaluated by detecting CD31-positive cells (clone SZ31, Dianova GmbH, Warburg, Hamburg, Germany) and by PAS staining (PAS Kit, Sigma-Aldrich) according to the manufacturers. Sections were scanned at 20× magnification.

#### Statistical analysis

Unless differently specified, for in vitro data at least three independent experiments in triplicate have been performed, and expressed as an average ± standard deviation. Linear regressions and correlation plots were computed via R scripting (specifically via lm and cor.test functions); results were considered to be statistically significant if *p* < 0.05. For in vivo experiments, each experimental group included eight mice. Two different experiments were performed. To determine the differences between tumor weights of different groups, Student’s *t*-test for unpaired data (two-sided) was used. Results were considered to be statistically significant if *p* < 0.05 after applying Student’s *t*-test.

## Electronic supplementary material

Supplementary Figure Legends

Supplementary Figures
