# Supplementary material for: BCL-XL overexpression promotes tumor progression-associated properties
Source: Cell Death Dis. 2017 Dec 13;8(12):3216. doi: 10.1038/s41419-017-0055-y (PMC5870591; doi:10.1038/s41419-017-0055-y)
Supplement: Supplementary file 1 — Supplementary Figure Legends [file 41419_2017_55_MOESM1_ESM.docx]

BCL-X_L_ overexpression promotes tumor progression-associated properties

Daniela Trisciuoglio*^1,5^, Maria Grazia Tupone*^1^, Marianna Desideri^1^, Marta Di Martile^1^, Chiara Gabellini^1°^, Simonetta Buglioni^2^, Matteo Pallocca^3^, Gabriele Alessandrini^4^, Simona D’Aguanno^§1^, Donatella Del Bufalo^§1^

^1^*Preclinical Models and New Therapeutic Agents Unit,* ^2^*Pathology Unit, ^3^SAFU Unit, ^4^Thoracic Surgery Unit, Regina Elena National Cancer Institute, Via Elio Chianesi 53, Rome (00144), Italy, ^5^Institute of Molecular Biology and Pathology, National Research Council, Via degli Apuli 4, Rome (00185), Italy.*

^§^Corresponding authors: Donatella Del Bufalo [donatella.delbufalo@ifo.gov.it](mailto:donatella.delbufalo@ifo.gov.it), Simona D’Aguanno [simona.daguanno@ifo.gov.it](mailto:simona.daguanno@ifo.gov.it), phone: +390652662575, fax: +390652662013

°Present address: Unit of Cell and Developmental Biology, Department of Biology, University of Pisa, S.S. 12 Abetone e Brennero 4, Pisa, Italy

*These authors equally contributed as first author

**Supplementary Figure legends**

**Figure S1**. **BCL-X_L_** **overexpression protects from drug-induced apoptosis in melanoma and glioblastoma models.** Percentage of cells in SubG1 phase evaluated by cytofluorimetric analysis of PI staining of melanoma control (Mneo) and BCL-X_L_ overexpressing (MXL90) cells (**a,c**), and in glioblastoma control (AN8) and BCL-X_L_ overexpressing (AXL74) cells (**b,d**) treated with two different concentrations of staurosporin (STR) or cisplatin (DDP) for 24 hours. (**e**) Western blotting analysis of PARP activation in Mneo and MXL90 cells treated with different concentrations of DDP alone and in combination with zVAD for 24 hours. HSP70/72 expression was evaluated to confirm equivalent transfer and loading. **(a,e)** Reported images/western blotting are representative of two independent experiments with similar results.

**Figure S2. Apoptosis was not activated in control cells during *in vitro* proliferation**. **a**) Flow cytometric quantification of apoptotic cells by AnnexinV-FITC/PI staining and **b**) Western blotting analysis of PARP activation in melanoma control (Mneo) and BCL-X_L_ overexpressing (MXL90) cells, glioblastoma control (AN8) and BCL-X_L_ overexpressing (AXL74) cells cultured for 6, 18, 24 and 120 hours. **(b)** HSP70/72 expression was evaluated to confirm equivalent transfer and loading. As positive control, parental M14 and ADF cells were exposed to 20µg/ml cisplatin cisplatin (DDP) for 24 hours. **(a,b)** Reported images/western blotting are representative of two independent experiments with similar results.

**Figure S3. BCL-X_L_ inhibition reduces *in vitro* cell migration and invasion, vasculogenic mimicry and spheroid forming ability of glioma cells.** (**a)** Quantification and representative images of *in vitro* cell migration and invasion of glioma BCL-X_L_ overexpressing (AXL74) cells exposed to WEHI-539 (20μM for 24 hours), a selective BCL-XL inhibitor. Values are expressed as percentage of migrated/invaded cells respect to control. (**b**) Quantification (tube length and number of intersection point) and representative images of capillary-like structure formation in AXL74 cells treated with WEHI-539 (20μM for 24 hours). (**c**) Quantification and representative images of tumor sphere forming capacity by AXL74 cells treated with 20μM WEHI-539. Data shown represent the fold of spheroids formation over control. (**a-c**) Data were expressed as average ± standard deviation. *p<0.05.

**Figure S4. Inhibition of BCL-X_L_ does not affect cell viability of spheroids.** Flow cytometric quantification of viable (AnnexinV-FITC negative) and apoptotic (AnnexinV-FITC positive) cells in (a) melanoma control (Mneo) and BCL-X_L_ overexpressing (MXL90) cells, cultured under spheroid condition for 10 days, (c) MXL90 transfected with siRNA oligonucleotides against BCL-X_L_ (si- BCL-X_L_) or scramble (si-Ctrl) target sequences, (d) MXL90 cells treated with 20μM WEHI-539. Data are expressed as percentage of apoptotic or viable cells. (b) Western blotting analysis of BCL-X_L_ protein expression in Mneo and MXL90 transfected with si-Ctrl and si- BCL-X_L_. After 48 h of transfection, cells were trypsinized and seeded under spheroid condition of growth. BCL-X_L_ protein expression was evaluated after 1, 5 and 9 days. β-actin expressions were evaluated to confirm equivalent transfer and loading. Western blotting analysis is representative of two independent experiments with similar results.

**Figure S5. Apoptosis-regulating genes are not modulated in melanoma and glioma models grown as 2D and 3D spheroid.** Quantitative Real-Time polymerase chain reaction analysis of BCL-X_L_, BCL-2, BAX, PUMA, NOXA, BIM mRNA in melanoma control (Mneo) and BCL-X_L_ overexpressing (MXL90) cells, and in glioblastoma control (AN8) and BCL-X_L_ overexpressing (AXL74) cells, grown (**a**) under 2D condition or (**b**) as 3D tumor spheroids. Two independent experiments were performed. Values are expressed as means of ratio ± standard deviation, where ‘ratio’ was calculated considering BCL-X_L_ overexpressing cells *versus* control cells, *p<0.05.

**Figure S6. Representative images of *in vitro* cell migration and invasion of melanoma (a,b) and glioma (c,d) cells with different levels of endogenous BCL-X_L_** **protein expression.**

**Figure S7. BCL-2** **modulates the formation of tumor spheroids.** (**a**) Western blotting analysis of BCL-2 expression in M14 melanoma control (puro) and BCL-2 overexpressing (BCL-2/6) cells. (**b**) Representative images of tumor sphere forming capacity of puro and BCL-2/6 cells. (**c,d,g**) Quantification of tumor sphere forming capacity by (**c**) puro and BCL-2/6 cells treated with 50µM zVAD or DMSO (Ctrl), (**d**) puro and BCL-2/6 cells treated with DMSO (Ctrl), or 1μM ABT-199, in presence or absence of 50µM zVAD, (**g**) BCL-2/6 cells transfected with siRNA oligonucleotides against BCL-2 (si-BCL-2) or scramble (si-Ctrl) target sequences. (**e**) Western blotting analysis of cleaved PARP protein expression in puro and BCL-2/6 cells grown under spheroid condition and treated with 1 μM ABT-199, alone or in combination with 50 μM zVAD. HSP70/72 expression was evaluated to confirm equivalent transfer and loading. (**f**) Western blotting analysis of BCL-2 expression in BCL-2/6 cells transfected with siRNA oligonucleotides against BCL-2 (si-BCL-2) or scramble (si-Ctrl) target sequences. (**a,e,f**) β-actin and HSP70/72 expression was evaluated to confirm equivalent transfer and loading. Images are representative of three independent experiments with similar results. (**c,d,g)** Data shown represent the fold of spheroids formation over control *, p<0.05 after applying Student’s t-test.

**Figure 8. BCL-X_L_** **overexpression in melanoma cells exposed to hypoxia increases the expression of VEGF and the activity of HIF-1.** **(a)** Western blotting analysis of HIF-1α, HIF-1β, and BCL-X_L_ expression in melanoma control (Mneo) and BCL-X_L_ overexpressing (MXL90) cells, cultured under normoxia or hypoxia. (**b**) VEGF secretion evaluated by ELISA in Mneo and MXL90 cells, cultured under normoxia or hypoxia. (**c**) VEGF promoter (VEGF1511, VEGF385) and HIF-1 transcriptional (HRE) activity in Mneo and MXL90 cells cultured under hypoxia. **(d)** Western blotting analysis of MMP2 activation in Mneo and MXL90 cells cultured under normoxia or hypoxia. Pro- and active- MMP2 forms were indicated. **(e)** Western blot analysis of BCL-XL protein expression in JR8 melanoma control (J8neo) and BCL-XL overexpressing (J8XL8 and J8XL10) cells. **(f)** VEGF secretion evaluated by ELISA in J8 neo, J8 XL8 and J8 XL10 cells cultured under normoxia or hypoxia. **(g)** Western blot analysis of HIF-1α and HIF-1β expression in J8 neo, J8 XL8 and J8 XL10 cells growth under normoxia or hypoxia. (**a,d,e,g**) Western blot analyses are representative of three experiments with similar results. β-actin, HSP70/72 or HSP90 expression was used for control transfer and loading. **(a,d)** number related to densitometric analysis values for protein expression after normalization are reported. (**b,c,f**) Experiments were conducted in triplicate. *p<0.05 after applying Student’s t-test. **(a-d,f,g)** Cells were exposed to normoxia (N) or hypoxia (H) for 18 hours.
