# Supplementary figures and images for: BCL-XL overexpression promotes tumor progression-associated properties
Source: Cell Death Dis. 2017 Dec 13;8(12):3216. doi: 10.1038/s41419-017-0055-y (PMC5870591; doi:10.1038/s41419-017-0055-y)

## Figure S1

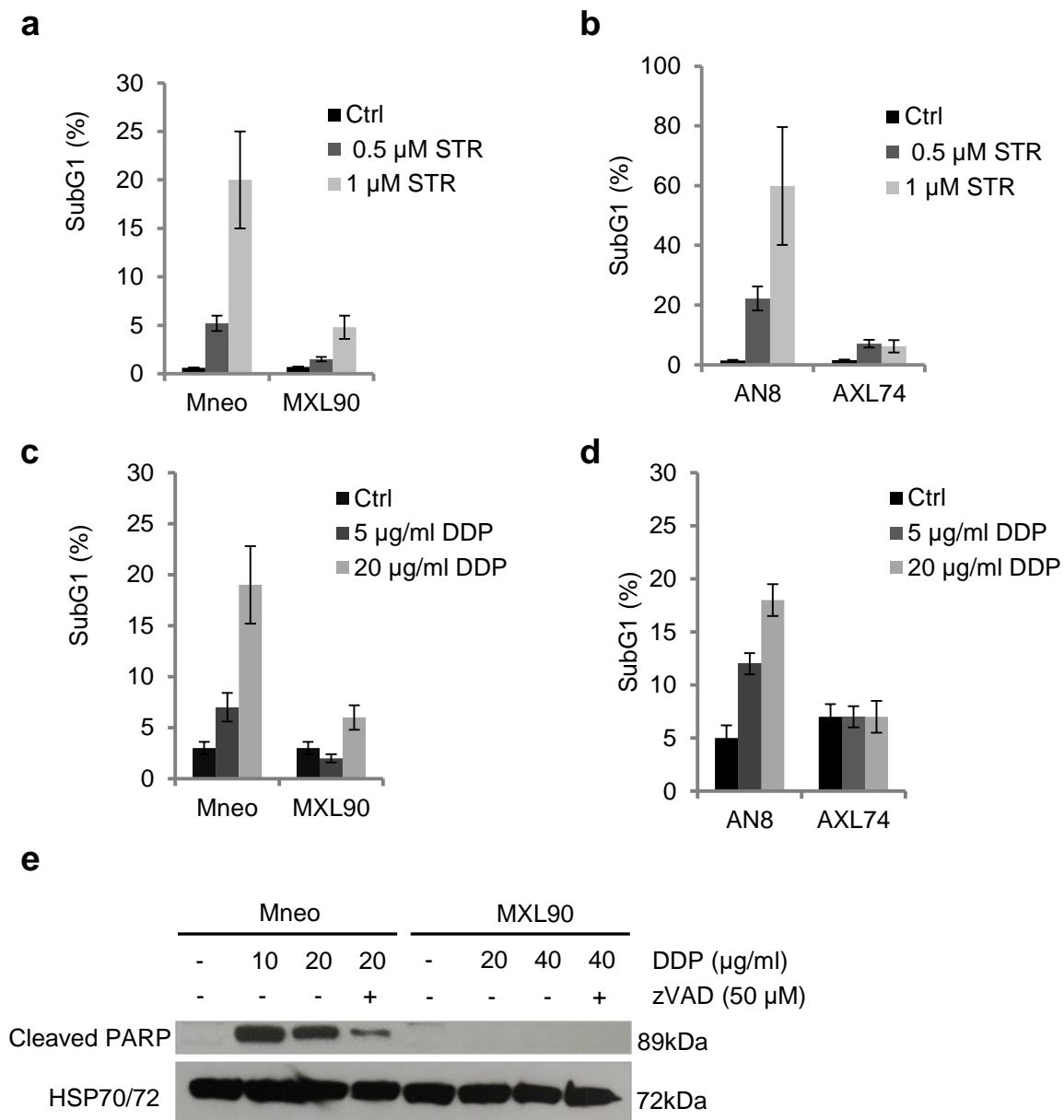

Figure S2

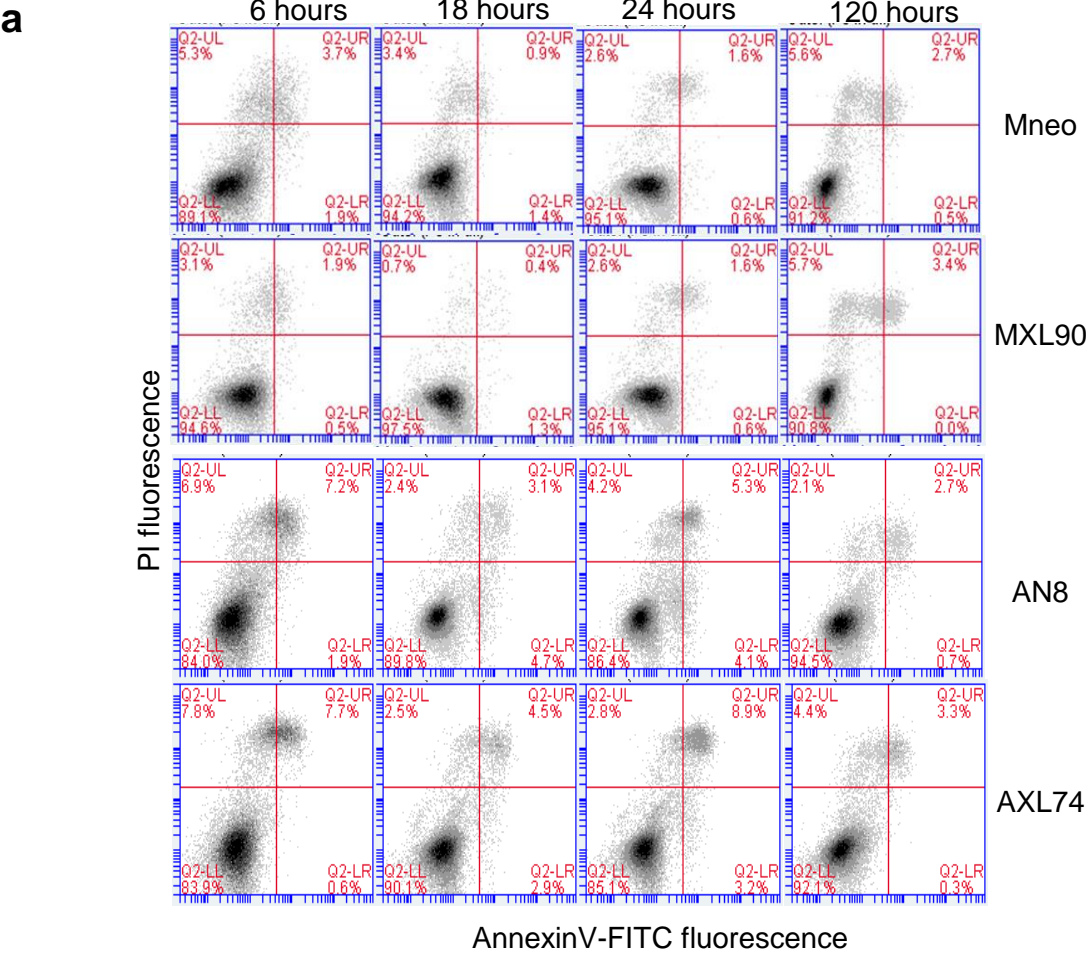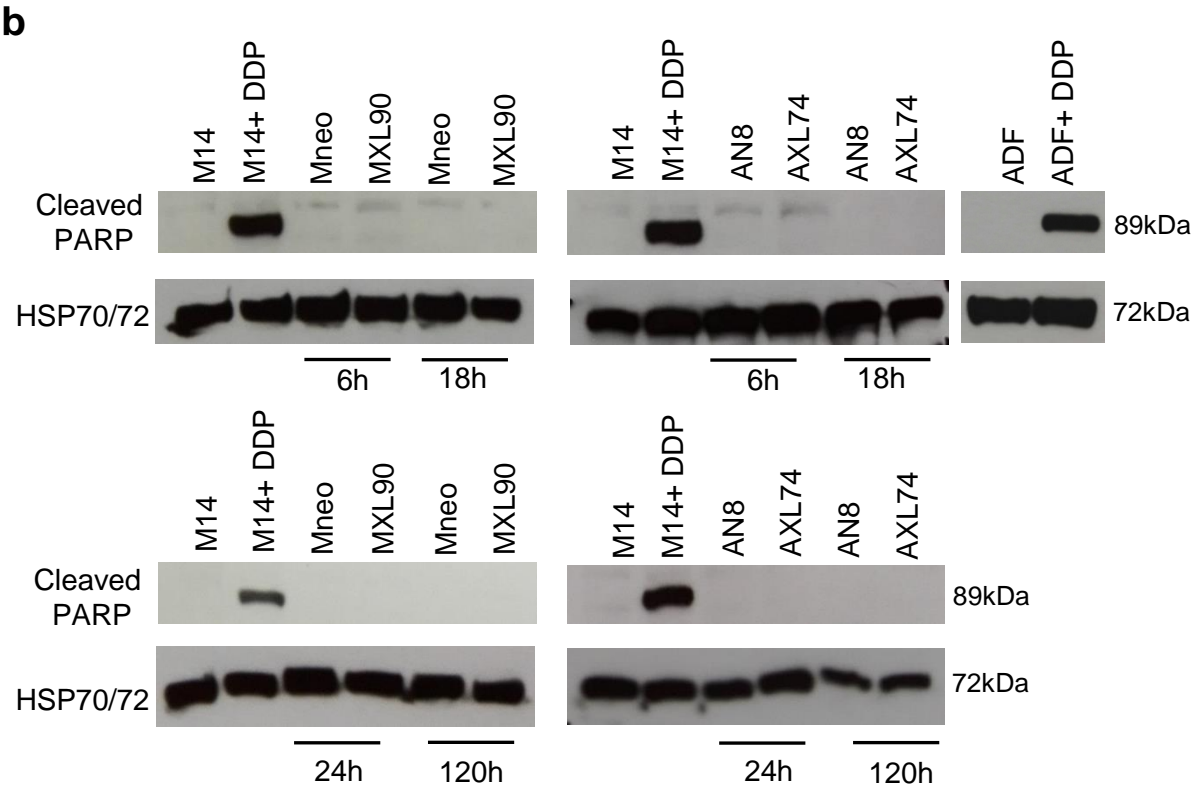

**Figure S3**

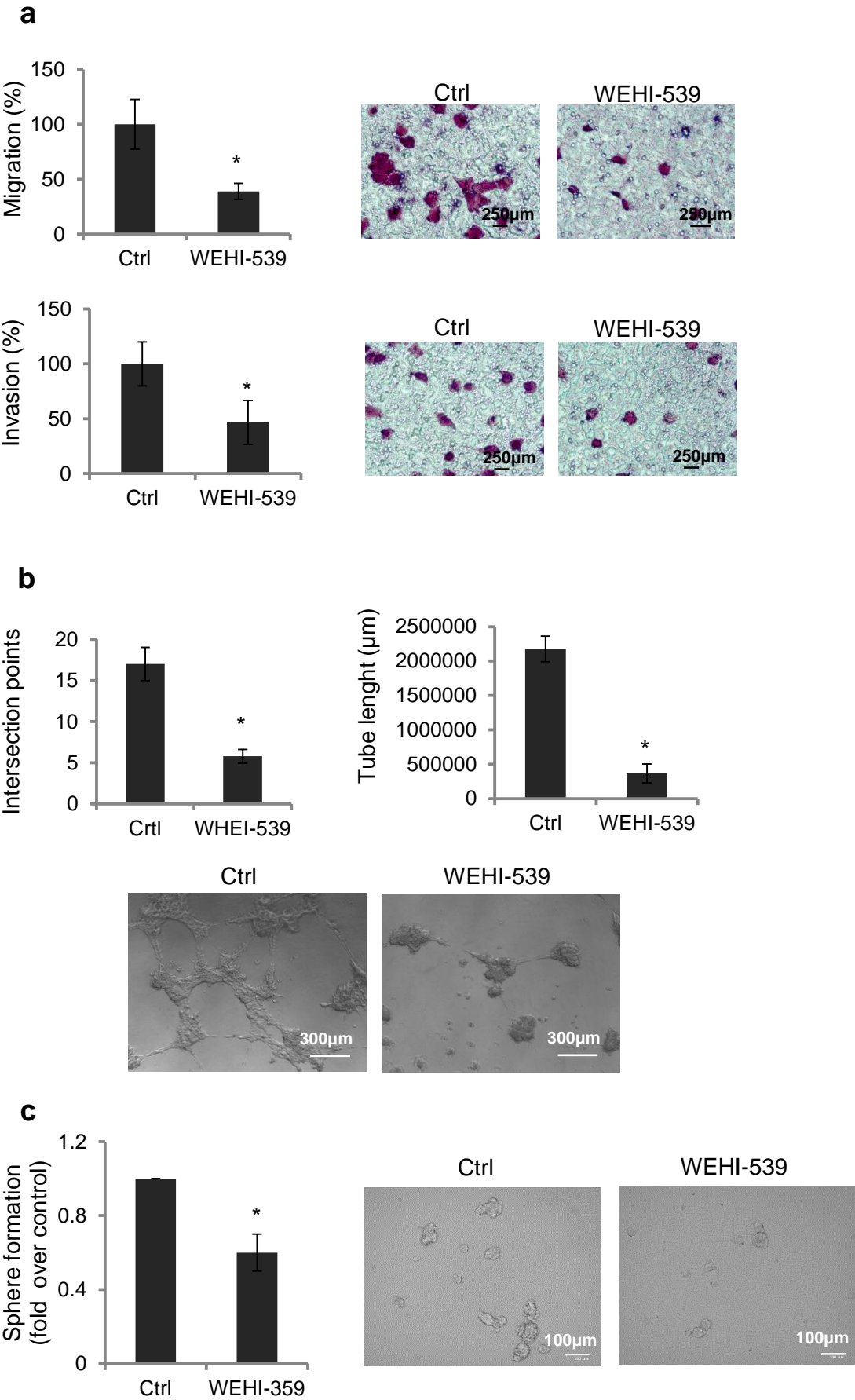

**Figure S4**

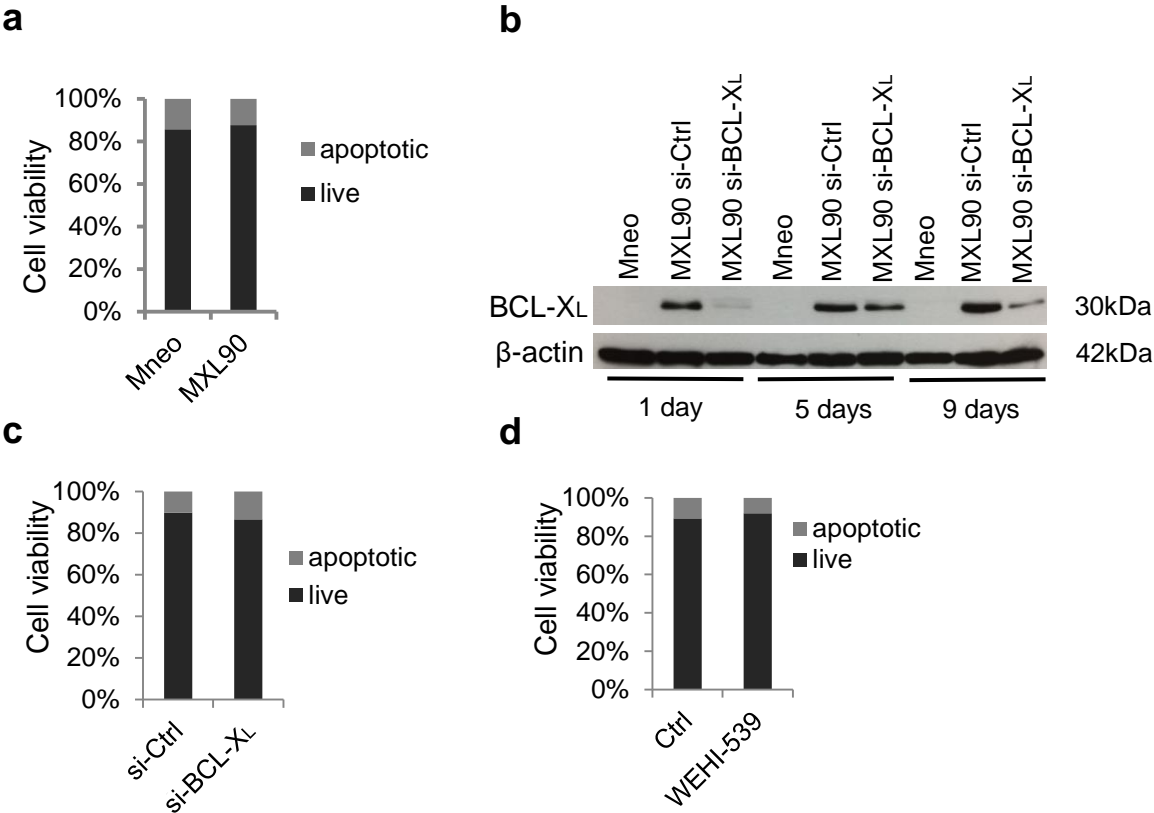

Figure S5

a

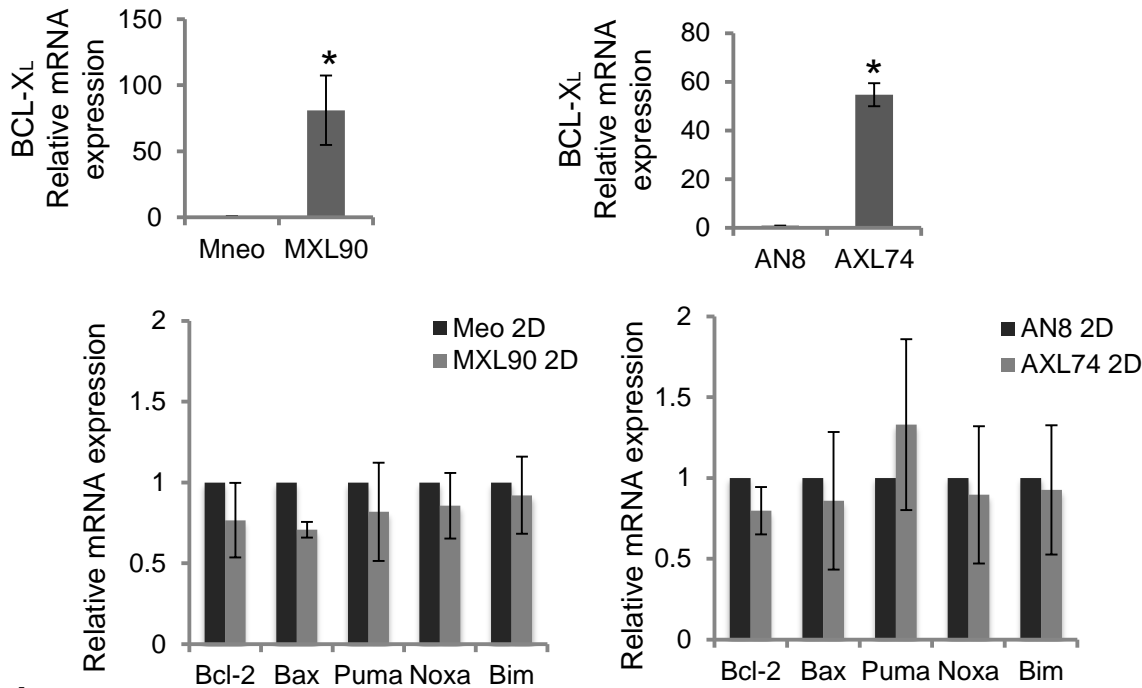

b

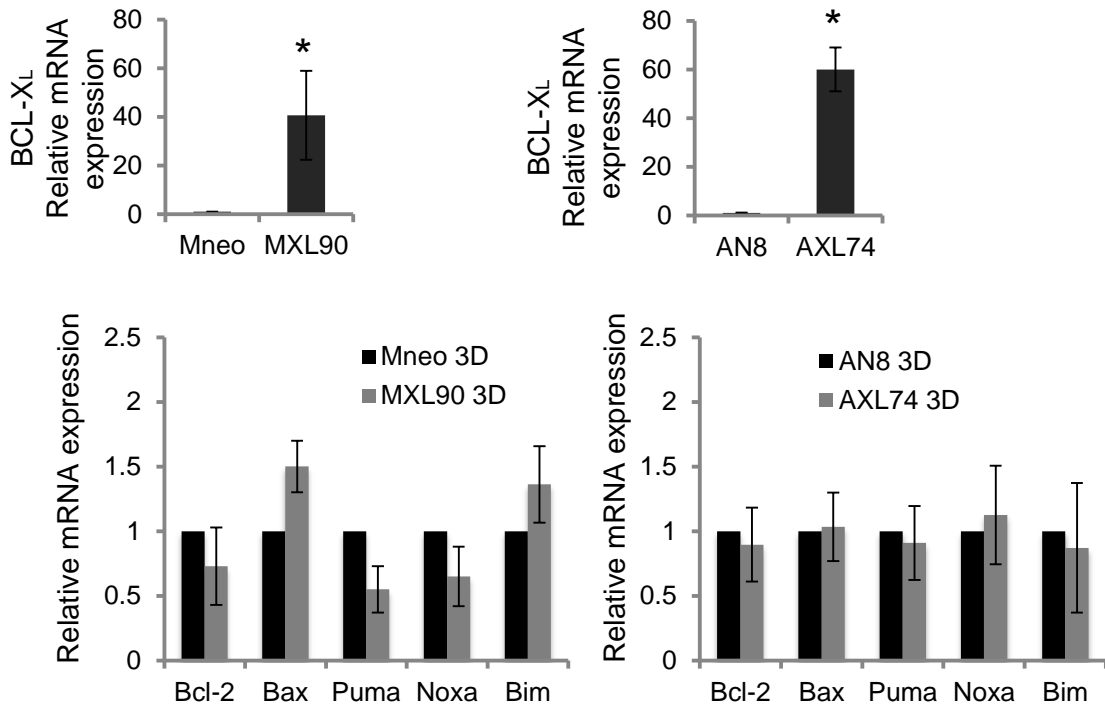

Figure S6

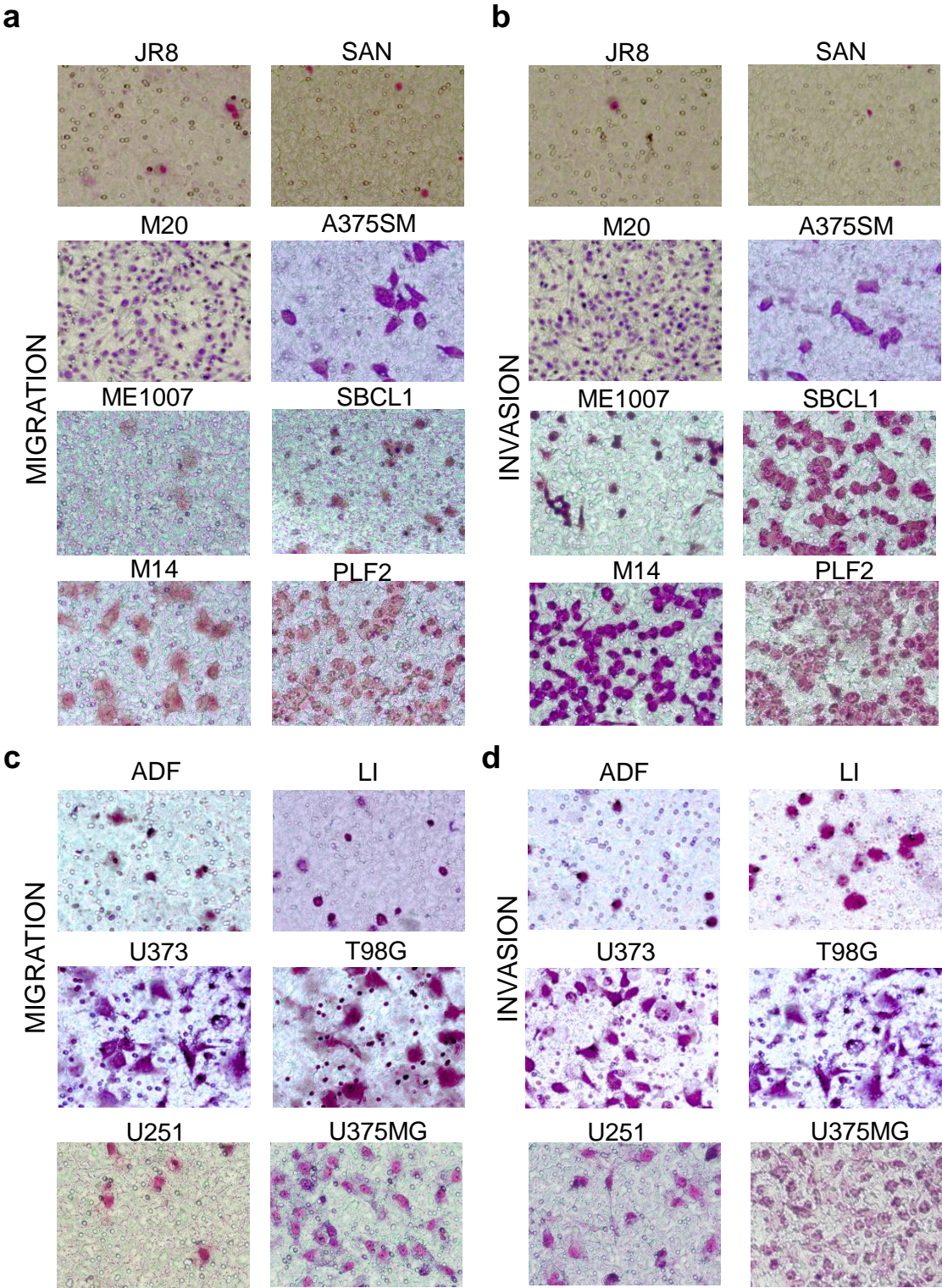

**Figure S7**

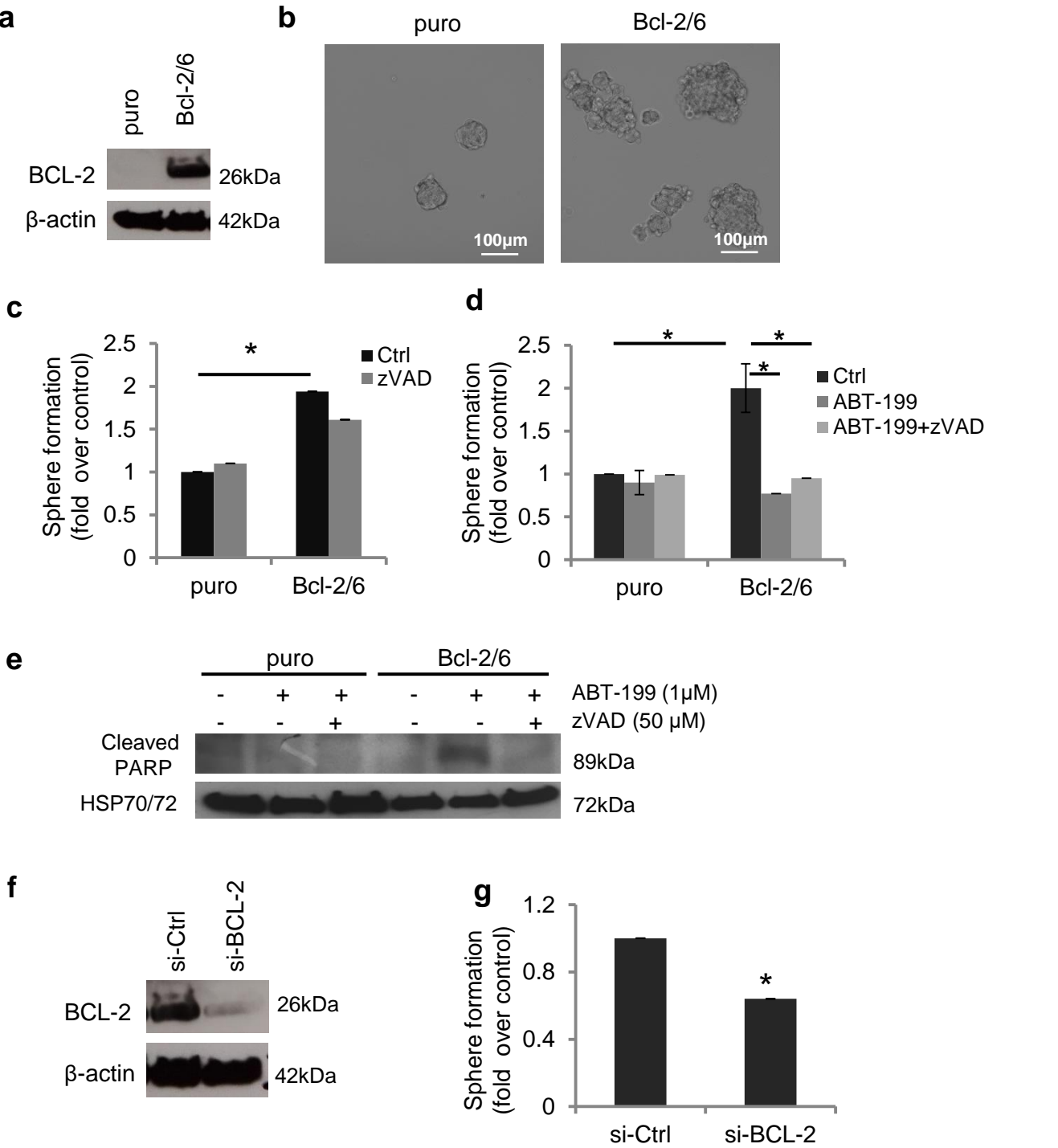

**Figure S8**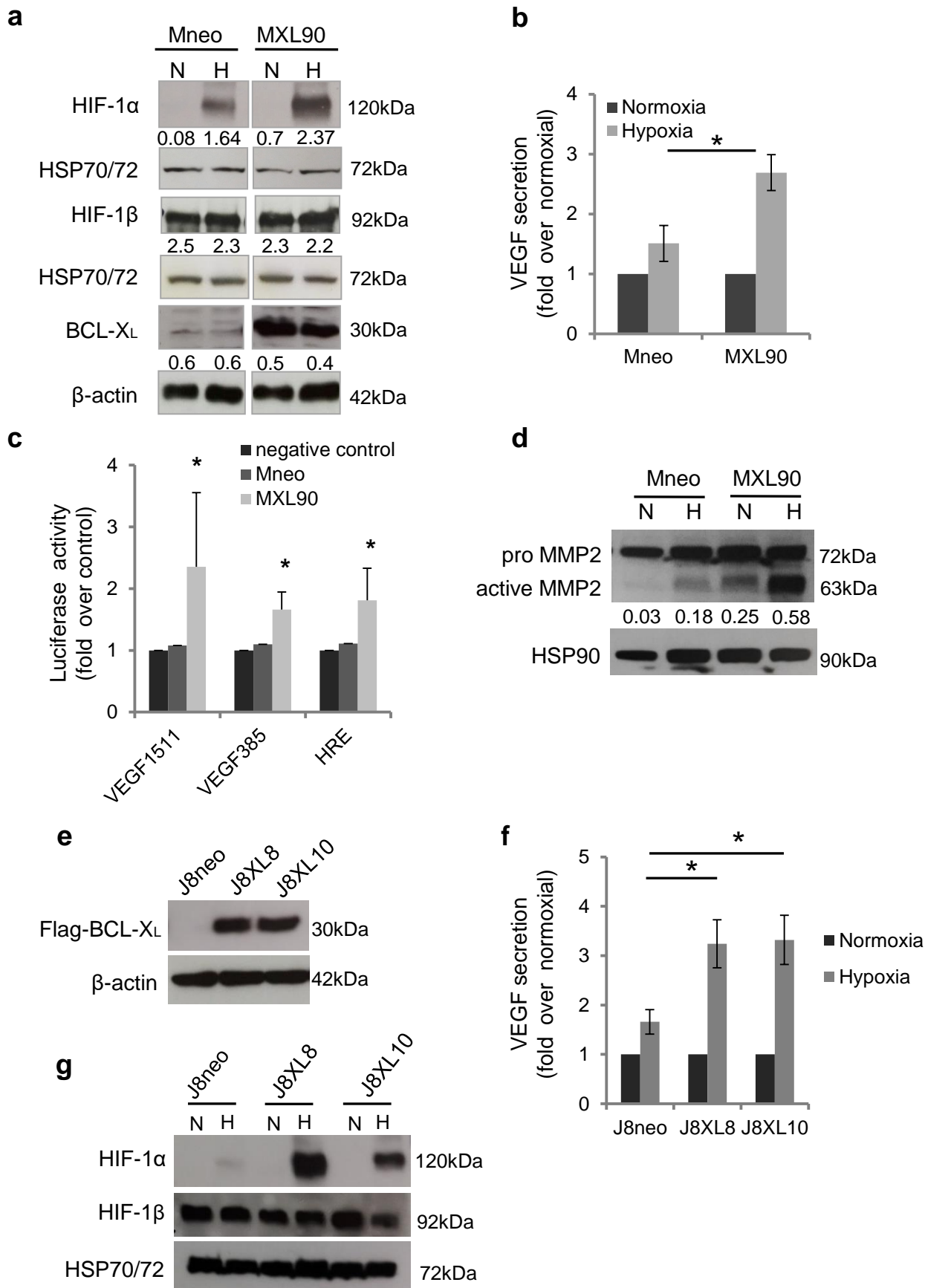

Supplement: Supplementary file 2 — Supplementary Figures [file 41419_2017_55_MOESM2_ESM.pdf]
